# The Antinociceptive Effects and Sex-Specific Neurotransmitter Modulation of Metformin in a Mouse Model of Fibromyalgia

**DOI:** 10.3390/cells13231986

**Published:** 2024-11-30

**Authors:** Hanin Abdulbaset AboTaleb, Hani A. Alturkistani, Gamal S. Abd El-Aziz, Emad A. Hindi, Mervat M. Halawani, Mona Ali Al-Thepyani, Badrah S. Alghamdi

**Affiliations:** 1 Department of Physiology, Faculty of Medicine, King Abdulaziz University, Jeddah 21589, Saudi Arabia; 2 Neuroscience and Geroscience Research Unit, King Fahd Medical Research Center, King Abdulaziz University, Jeddah 21589, Saudi Arabia; eahindi@kau.edu.sa (E.A.H.); mhalwani@kau.edu.sa (M.M.H.); mahalthepyani@kau.edu.sa (M.A.A.-T.); 3 Department of Clinical Anatomy, Faculty of Medicine, King Abdulaziz University, Jeddah 22252, Saudi Arabia; hturkustani@kau.edu.sa (H.A.A.); dr_gamal_said@yahoo.com (G.S.A.E.-A.); 4 Department of Chemistry, College of Sciences & Arts, King Abdulaziz University, Rabigh 21911, Saudi Arabia

**Keywords:** fibromyalgia, metformin, chronic pain, antinociceptive, neurotransmitters, histological changes, IL-1β, serotonin, norepinephrine

## Abstract

Fibromyalgia (FM) is a chronic and debilitating condition characterized by diffuse pain, often associated with symptoms such as fatigue, cognitive disturbances, and mood disorders. Metformin, an oral hypoglycemic agent, has recently gained attention for its potential benefits beyond glucose regulation. It has shown promise in alleviating neuropathic and inflammatory pain, suggesting that it could offer a novel approach to managing chronic pain conditions like FM. This study aimed to further explore metformin’s analgesic potential by evaluating its effects in an experimental FM model induced by reserpine in both male and female mice. After the administration of 200 mg/kg metformin to male and female mice, the FM-related symptoms were assessed, including mechanical allodynia, thermal hyperalgesia, and depressive-like behaviors. A histological examination of the thalamus, hippocampus, and spinal cord was conducted using haematoxylin and eosin staining. The neurotransmitter and proinflammatory cytokines levels were measured in the brains and spinal cords. Our results have shown that metformin treatment for seven days significantly reversed these FM-like symptoms, reducing pain sensitivity and improving mood-related behaviors in both the male and female mice. Additionally, metformin exhibited neuroprotective effects, mitigating reserpine-induced damage in the hippocampus, thalamus, and spinal cord. It also significantly lowered the levels of the proinflammatory cytokine interleukin 1-beta (IL-1β) in the brain and spinal cord. Notably, metformin modulated the neurotransmitter levels differently between the sexes, decreasing glutamate and increasing serotonin and norepinephrine in the male mice, but not in the females. These findings underscore metformin’s potential as an alternative therapy for FM, with sex-specific differences suggesting distinct mechanisms of action.

## 1. Introduction

Fibromyalgia (FM) is a chronic condition characterized by widespread pain, often accompanied by symptoms such as depression, sleep disturbances, fatigue, and cognitive dysfunction [1]. FM affects approximately 3–9% of the global population and is significantly more prevalent in females than in males, with a female-to-male ratio of 3:1 [2]. This condition is a major cause of disability, impacting patients’ quality of life and daily functioning [3,4]. Currently, there are no universally effective treatments for FM, largely due to the lack of consensus on its etiopathogenesis [3,4]. Since the 1990s, scientists have recognized a potential link between FM and neurohormonal alterations [5]. Central pain processing dysfunctions and neuroinflammation are also reported as plausible contributors to generalized pain sensitization in FM patients [6]. Additionally, FM patients exhibit reduced activity in their descending pain modulatory system across several brain regions, including the rostral anterior cingulate cortex, thalamus, periaqueductal gray, and rostral ventromedial medulla [7]. Animal studies further suggest that an imbalance—characterized by increased glutamatergic and decreased Gamma-aminobutyric acid (GABA)-ergic neurotransmission in the insular cortex or anterior cingulate cortex—may drive heightened pain sensitivity by intensifying central pain processing in FM [8]. Consequently, disruptions in neurotransmitter levels are now recognized as key elements in the pathophysiology of FM [9]. Despite these insights, the Food and Drug Administration (FDA) has endorsed three drugs for FM treatment: the antiseizure drug pregabalin and the antidepressants duloxetine and milnacipran [10]. Unfortunately, these drugs often achieve minimal pain reduction, and not all patients experience substantial pain relief [11]. Moreover, the side effects of these drugs sometimes outweigh their benefits [12,13,14,15]. Although lethal side effects such as heart failure, hepatic failure, or serotonin syndrome are rare, they must still be considered [11]. For these reasons, preclinical studies are crucial in explaining the pathophysiology of FM and identifying new therapeutic targets. These studies are essential for exploring innovative treatment options, particularly given the limitations in efficacy associated with current therapeutic approaches. 

Metformin is a hypoglycemic agent that has been widely used as a first-line treatment for type 2 diabetes [16]. Recent research has unveiled a novel relationship between metformin and pain management, suggesting that metformin may serve as a promising antinociceptive agent [17]. It has been found to have valuable impacts on various persistent pain pathologies, such as complex regional pain syndrome, neuropathic pain, diabetic neuropathy, and FM-type pain [18,19,20,21]. Interestingly, metformin has also been shown to alleviate common associative symptoms of chronic pathological pain, including depression, anxiety, and cognitive impairment, in both rodents and humans [20]. Notably, recent studies suggest that metformin can improve serotonin and norepinephrine levels in the brain [22,23]. Additionally, metformin administration has been shown to normalize the brain glutamate levels in diabetic epileptic rodent models [24]. Moreover, FM is frequently associated with overweight, obesity, and related metabolic disturbances, which may exacerbate symptom severity and adversely affect one’s quality of life. Given metformin’s known effects on these parameters, it presents intriguing potential as a therapeutic agent for FM [25]. 

While preclinical studies have shown the promising benefits of metformin in some chronic pain conditions, research on its antinociceptive effects in FM-like pain remains limited. This study aims to fill this gap by examining the antinociceptive effects of metformin on an experimental FM model. We hypothesize that metformin exhibits antinociceptive effects in a FM mouse model by alleviating pain sensitivity and improving mood-related behaviors through modulating neurotransmitter levels, reducing neuroinflammation, and protecting the neuronal structure in the brain and spinal cord. Therefore, this research aims to assess the influence of metformin on FM symptoms and explore its therapeutic potential in addressing certain aspects of FM-related pathophysiology. This study included male and female mice to identify any potential sex differences in response to treatment that could help guide more personalized treatments for FM in the future. 

## 2. Materials and Methods

### 2.1. Animals 

This study involved a total of 104 Swiss albino mice (30–40 g, 9–10 weeks old), consisting of 52 males and 52 females. All the animals were sourced from the King Fahad Medical Research Center in Jeddah, Saudi Arabia. A maximum of five mice were housed in clear, transparent polycarbonate cages with straw bedding, which was regularly changed. The mice had constant access to food and water and were kept on a standard 12 h light/dark cycle at a room temperature of 23 ± 2 °C. The animals were acclimatized to the laboratory environment for four days before the start of the experiments. Behavioral evaluations were conducted between 9:00 a.m. and 2:00 p.m. This study followed the ethical guidelines set by the Biomedical Ethics Committee of King Abdulaziz University (approval no. 236-24) and the Animal Care and Use Committee (ACUC) of the Animal House Unit, King Fahad Medical Research Center, Jeddah, Saudi Arabia.

### 2.2. Drugs

Reserpine (98 %; Acros Organs—Thermo Fisher Scientific, Waltham, MA, USA), metformin hydrochloride (Solarbio Science & Technology Co., Ltd., Beijing, China), and pregabalin (Saudi Pharmaceutical Industries, Jeddah, Saudi Arabia) were used in this study. The reserpine was diluted to its final concentration in 0.5% glacial acetic acid (*v*/*v* in distilled water) and administered subcutaneously to the mice at a volume of 6.3 mL/kg. The FM-like state was induced in the mice by injecting reserpine (0.5 mg/kg/day) for three consecutive days, following the method described in the literature [26,27]. We selected this dosing regimen based on a preliminary study to determine the most appropriate dose of reserpine for inducing FM-like symptoms in our specific mouse strain [28]. Solutions of metformin and pregabalin were prepared daily in sterile saline and administered to the mice intraperitoneally at a volume of 10 mL/kg. Pregabalin was used as a positive control drug, and it was administered to the mice at the same schedule of treatment. The doses of metformin (200 mg/kg) and pregabalin (30 mg/kg) were based on established protocols from previous research [18,29].

### 2.3. Treatment and Assessment Strategy

Animals were randomly assigned into four groups: the vehicle control group, the RES + saline group, the RES + metformin group, and the RES + pregabalin group. For the first 3 days of the experiment, the vehicle control group received subcutaneous injections of vehicle solution (0.5% glacial acetic acid in distilled water), and the other groups received subcutaneous injections of reserpine. Beginning on day 4 and continuing through day 10 of the experiment, the mice received daily intraperitoneal injections of saline, metformin, or pregabalin.

**Figure 1:**
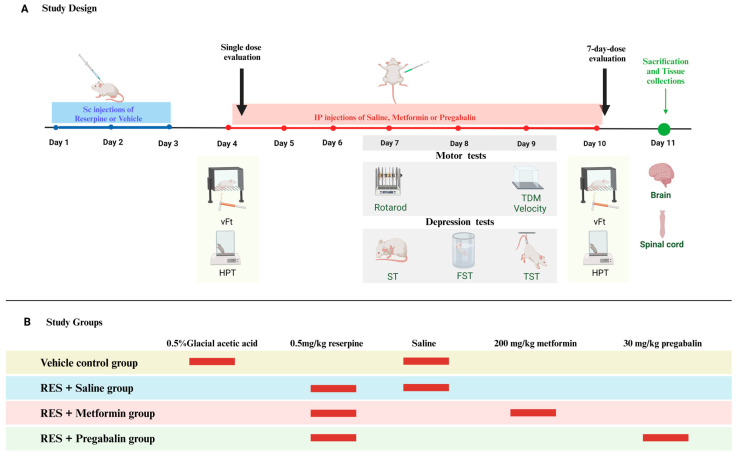
Schematic representation of the overall design and timeline of the study. (**A**) Total duration of the study was 11 days. Reserpine (RES) was administered subcutaneously (Sc) to the mice during the first 3 days. Behavioral tests were performed on days 4, 7, 8, 9, and 10. On day 11, the mice were sacrificed, and brain and spinal cord samples were collected for histopathological and biochemical analysis. (**B**) Study groups and the agents they received. Abbreviations: vFt, von Frey test; HPT, hot plate test; TDM, total distance moved; FST, forced swimming test; TST, tail suspension test; ST, splash test; IP, intraperitoneal injection; Sc, subcutaneous injection. Created in BioRender. AboTaleb, H. https://BioRender.com/z08v394 (1 January 2024).

A sensory behavioral analysis was performed on the 4th and 10th days of the study to evaluate the effects of single and 7-day treatments, respectively. The drug was injected first, and sensory tests were conducted 3 h post-injection. Motor behavioral analyses were carried out on the 7th and 9th days, while depression-related behaviors were assessed on the 7th, 8th, and 9th days. To minimize any potential influence of drug injections on the results, injections were administered after the completion of the motor and depression tests. We conducted behavioral tests in the middle of the study, ensuring that each group of mice was tested only once per day for each type of assessment. On day 11, the animals were dissected, and brain and spinal cord samples were collected for biochemical and histological evaluations. Figure 1 displays the timeline of this study.

### 2.4. Assessment of the Pain Threshold

#### 2.4.1. Von Frey Test (vFt)

The mechanical thresholds were measured by using von Frey filaments following the up-down paradigm, as described in the literature [30]. The mice were positioned individually in clear plexiglass boxes on the elevated mesh platform measuring 90 × 38 cm, with a grid of square holes approximately 5 × 5 mm. The filaments (the Aesthesio^®^ set, UGO-37450-275) with increasing stiffness (0.04–4 g) were employed to the plantar surface of the left hind paw, and the responses were recorded in an XO pattern. Paw lifting was recorded as (X), indicating a positive response, while no response within 5 s was recorded as (O). The stimulation began with 0.6 g and continued until the completion of five succeeding positive responses (assigned a score of 0.04), five consecutive negative responses (assigned a score of 4), or four readings following the first different response. The threshold for paw withdrawal was measured in grams (g) and calculated using the threshold calculator website designed by Christensen SL and his groups [31]. A noticeable drop in the paw withdrawal threshold relative to the control values was considered mechanical allodynia [32]. The mice were habituated to the mesh platform for at least 45 min before testing.

#### 2.4.2. Hot Plate Test

A hot plate apparatus (Ugo Basile, Gemonio, Italy) was used to assess the thermal threshold in the mice as described previously [33]. The temperature of the hot plate’s surface was kept constant at 55 ± 0.1 °C [34]. The mice then were positioned on the heated metal plate that enclosed via a clear acrylic cylinder. To avoid causing any tissue damage, the latency to heat response, indicated by flicking, hind paw licking, or jumping, was recorded with a 30 s cut-off period [35].

### 2.5. Assessment of Motor Behavior 

#### 2.5.1. Open Field Test (OFT)

On the 9th day of the experiment, each mouse was positioned in the center of an acrylic box arena (45 cm × 45 cm × 34 cm) and given 3 min to move around freely in a sound-attenuated room, under low-intensity light. The movement of the mice, including their total distance moved (TDM) in centimeters and the velocity in centimeters per sec (cm/s), was tracked and recorded using the EthoVision XT8A system (Noldus Information Technology, Wageningen, The Netherlands) [36].

#### 2.5.2. Rotarod Test 

This study aimed to examine the effects of different interventions on fatigue and muscle coordination using a rotarod test, which is also recommended for assessing nociception in rodents [37]. The mice were trained on a rotarod at a speed of 9 rpm until they could remain on the rotating device for 30 s without falling. A total of 1 h later, their ability to stay on the rotarod at a fixed speed of 20 rpm was recorded for up to 240 s [38,39]. This procedure was repeated for three trials at 10 min intervals, and then the average performance was calculated. The rotarod test was conducted on the 7th day of the study. 

### 2.6. Measurement of Depressive-like Behavior

#### 2.6.1. Splash Test (ST)

An ST was performed to measure anti-anhedonia-like behavior, which is one of the major signs of depression [40]. In this test, self-cleaning behavior was assessed by squirting a sucrose solution (300 μL of 10%) onto each mouse’s dorsal coat and recording its grooming behavior for 5 min [7]. The grooming behavior was recorded by either licking or scratching the mice at their body or limbs, particularly in the areas affected by the splash solution. The ST was conducted on the 7th day of the study.

#### 2.6.2. Forced Swimming Test (FST)

An FST was used to identify potential depressive-like behavior in the experimental animals [41]. The test was performed following the method described in the literature [42]. In this test, each mouse was placed in a separate glass cylinder (10 cm in diameter and 20 cm in height) filled with water maintained at 23–25 °C, and allowed to swim for 6 min. The immobility of the mice during the last 4 min was recorded in sec [34]. The absence of paw movements other than those required to maintain the mouse’s head above water was defined as immobility [27]. The FST was performed on the 8th day of the study.

#### 2.6.3. Tail Suspension Test (TST)

To evaluate depression-like behavior, TSTs have been commonly utilized [43]. In this test, the mice were suspended by their tails approximately 50 cm above the bench, with their tails adhered to the wall using adhesive tape and a barrier to obstruct the mouse’s view. The suspension lasted for 6 min, and the total immobility time was evaluated during the last 4 min. The mice were considered immobile when they stopped struggling to escape the suspended position [44]. The TST was performed on the 9th day of the study.

### 2.7. Tissue Harvesting and Processing 

At the end of the study, the mice were killed via cervical dislocation, and their brains and spinal cords were harvested. Four samples from each group were immediately fixed in 10% formalin and then processed for histopathological analysis within 48 h. The remaining samples were stored at −80 °C for further biochemical analysis.

#### 2.7.1. Histopathological Analysis

The standard protocol described in Alqurashi et al. 2022 was adhered to for performing hematoxylin and eosin (H&E) staining [44]. Briefly, after 16 h of tissue processing using the Spin Tissue Processor STP120 (Especialidades Médicas Myr, S.L., Tarragona, Spain), the midsagittal hemisected brains were immersed in paraffin wax and sectioned into 4 µm-thick slices using microtomes (LEICA RM 2255, Leica Microsystems, Germany). To characterize the histopathological changes, the slides were stained with H&E using the Myr AutoStainer (Especialidades Médicas Myr, S.L., Tarragona, Spain) and examined under an Olympus BX53 light microscope to assess the alterations in the hippocampus, thalamus, and spinal cord. The representative areas were captured at ×100, ×200, and ×400 magnifications using an Olympus DP73 camera and the images were processed using Olympus CellSens Entry software (Olympus Corporation, Tokyo, Japan).

#### 2.7.2. Biochemical Analysis

The minced brain and spinal cord tissues were weighed and added to a homogenization buffer containing 0.25 M sucrose, 1 mM EDTA, 5 mM MOPS, and 0.1% ethanol, with the pH adjusted to 7.2 using 1 M NaOH. The tissues were prepared at a ratio of 0.1 of tissue per 1 mL of solution. The tissue was thoroughly homogenized and then centrifuged at 1000 *g* for 10 min at 4 °C. The resulting supernatant was collected for the subsequent estimation of the neurotransmitter and proinflammatory cytokine levels.

#### 2.7.3. Measurement of Serotonin and Norepinephrine Levels

The serotonin and norepinephrine levels were determined using ELISA kits (SEKSM-0016 and SEKSM-0019, respectively) obtained from Solarbio, in accordance with the manufacturer’s instructions (Solarbio Science & Technology Co., Ltd., Beijing, China). Briefly, the test procedure involved adding 50 µL of samples and standards to each well of a microplate, followed immediately by 50 µL of a working solution of a biotin-conjugated anti-ST/5-HT or anti-NA/NE antibody. At 37 °C, the plate was incubated for 45 min. Upon cleaning the plate, 100 µL of a streptavidin-labeled detection antibody was introduced and incubated for another half hour at 37 °C. After aspirating and washing it three times, 90 µL of substrate solution was added, followed 30 min later by 50 µL of stop solution. The optical absorbance of each well was then immediately measured at 450 nm using a microplate reader (BioTek Instruments, Inc., Winooski, VT, USA), and the absorbance measurements were compared to the calibration plot for standard solutions to determine the readings. 

#### 2.7.4. Measurement of Glutamic Acid Levels

The glutamate levels were assessed using the Glutamic Acid Content Assay kit (WST-1 chromogenic method) obtained from Solarbio, as per the manufacturer’s instructions (Solarbio Science & Technology Co., Ltd., Beijing, China). The absorbance values were measured at 450 nm using a microplate reader (BioTek Instruments, Inc., Winooski, VT, USA), and the results were established by comparing these values with the calibration plot for standard solutions.

#### 2.7.5. Measurement of TNF-α and IL-1β Levels

The levels of IL-1β and TNF-α were determined using ELISA kits (SEKM-0002 and SEKM-0034, respectively) from Solarbio following the manufacturer’s instructions (Solarbio Science & Technology Co., Ltd., Beijing, China). Briefly, the assay procedure involved adding 100 µL of the samples and standards to the microplate wells and incubating them at 37 °C for 90 min. After washing the plate, 100 µL of biotin-conjugated anti-mouse IL-1β or TNF-α antibody working solution was added to each well. The plate was incubated at 37 °C for 60 min. After washing the plate again, 100 µL of the streptavidin-labeled detection antibody was added to the microplate wells and incubated again at 37 °C for 30 min. After aspirating and washing it three times, 100 µL of substrate solution was added. After 15 min, 50 µL of stop solution was added, and the optical absorbance of each well was immediately read at 450 nm using a microplate reader (BioTek Instruments, Inc., Winooski, VT, USA). The concentrations were determined by comparing the absorbance values with the calibration chart for standard solutions.

### 2.8. Statistical Analysis 

Statistical analyses were performed using GraphPad PRISM software for Windows (version 10, GraphPad Software, San Diego, CA, USA). The data were displayed as the mean ± standard error of the mean (SEM), and the figure legends included the number of mice or samples used in each analysis. The normality was assumed using a Shapiro–Wilk’s test. The differences between the groups were analyzed using an ordinary one-way ANOVA followed by Tukey’s multiple comparison test, or Kruskal–Wallis tests as appropriate. A *p*-value of less than 0.05 was considered statistically significant. 

## 3. Results

### 3.1. Metformin Treatment Reversed Reserpine-Induced Mechanical Allodynia and Thermal Hypersensitivity in Male and Female Mice After 7 Days of Treatment

The vFt is a valuable tool for understanding pain mechanisms and evaluating potential therapies in research on pain disorders [35]. To evaluate the effects of single and continuous 7-day metformin injections on established reserpine-induced mechanical allodynia in male and female mice, the responses were assessed using the vFt. Starting on day 4 of the study, the mice received intraperitoneal injections of metformin (200 mg/kg), pregabalin (30 mg/kg), or saline.

**Figure 2:**
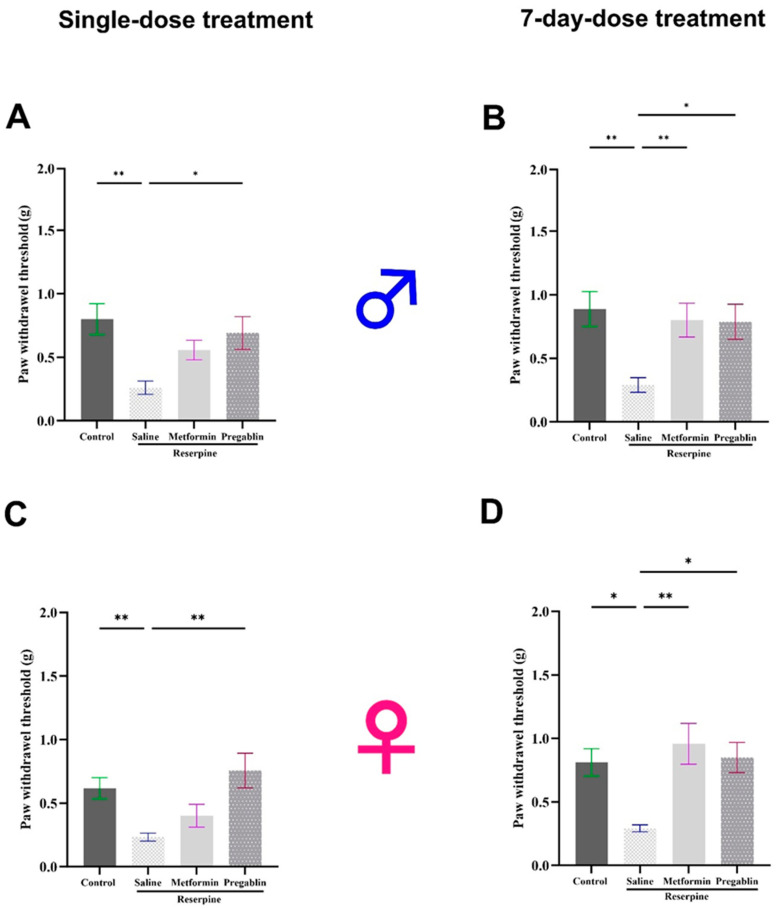
Effect of different interventions on mechanical allodynia in male and female mice tested after single and 7-day treatment. (**A**,**B**) Paw withdrawal threshold in von Frey test in male mice. Reserpine administration (0.5 mg/kg) reduced paw withdrawal threshold on all tested days. A single dose of metformin (200 mg/kg) did not reverse the effects of reserpine on mechanical threshold, whereas 7-day dosing significantly alleviated mechanical allodynia. (**C**,**D**) Paw withdrawal threshold in von Frey test in female mice. Single dose of metformin had no effect, while 7-day dosing significantly alleviated mechanical allodynia. Pregabalin (30 mg/kg), used as a positive control, effectively restored paw withdrawal threshold toward control levels across all testing days in both male and female mice. Each bar represents mean, and vertical lines indicate standard error mean (SEM) for 7–12 mice/group. Asterisks above lines indicate significant difference between groups where * *p* < 0.05 and ** *p* < 0.01, otherwise, a non-significant difference is recorded.

In the male mice, reserpine administration significantly decreased the mechanical nociceptive thresholds at days 4 and 10 of the study compared to the vehicle control group (*p* = 0.0030 and *p* = 0.0021, respectively). Seven days of metformin injections reversed the reserpine-induced mechanical allodynia (*p* = 0.0061), whereas a single metformin injection did not produce a significant acute effect (Figure 2A,B). 

Similar results were observed in the female mice, where subcutaneous injections of reserpine significantly reduced the paw withdrawal threshold compared to the control mice receiving the vehicle on days 4 and 10 (*p* = 0.0027 and *p* = 0.0482, respectively). Seven days of metformin treatment significantly reduced reserpine-induced mechanical allodynia compared to the saline-treated mice (*p* = 0.0044). However, it did not produce a significant acute effect after a single dose (Figure 2C,D).

In contrast to the effects of metformin, a single pregabalin injection significantly increased the paw withdrawal threshold compared to the saline group in both the male (*p* = 0.0271) and female mice (*p* = 0.0013).

**Figure 3:**
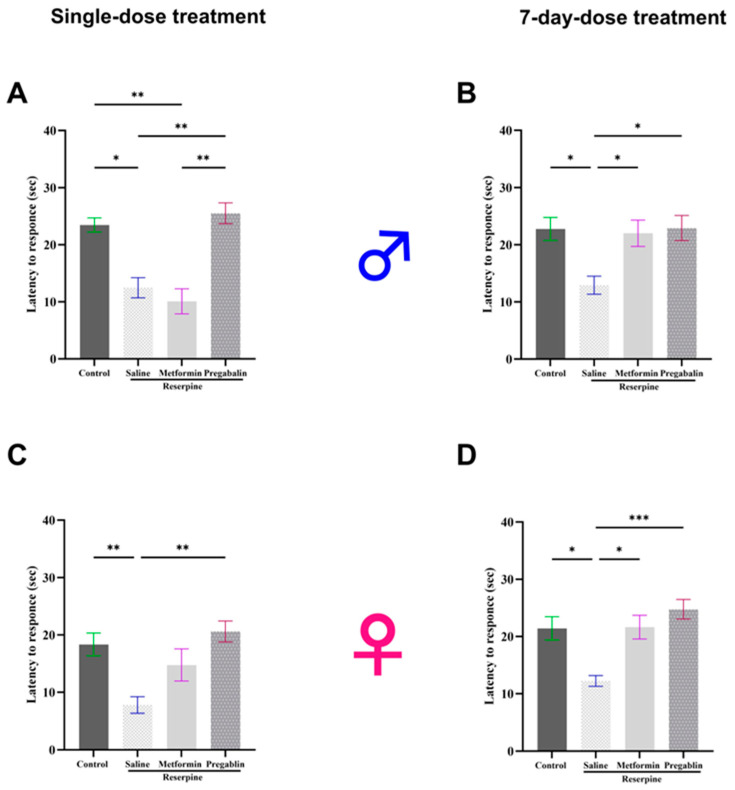
Effect of different interventions on thermal hypersensitivity in male and female mice tested after single and 7-day treatment. (**A**,**B**) Latency to respond in hot plate test in male mice. Reserpine administration (0.5 mg/kg) reduced latency of response to hot stimuli on all tested days. Single dose of metformin (200 mg/kg) did not reverse effects of reserpine on hot threshold, while 7-day dosing significantly alleviated thermal hypersensitivity. (**C**,**D**) Latency to respond to hot stimulus in female mice. Single metformin dose had no effect, while 7-day dosing significantly alleviated thermal hypersensitivity. Pregabalin (30 mg/kg), used as a positive control, effectively restored latency to respond to hot stimulus toward control levels across all testing days in both male and female mice. Each bar represents mean, and vertical lines indicate standard error mean (SEM) for 8–13 mice/group. Significant difference between groups is indicated by asterisks above the lines where * *p* < 0.05, ** *p* < 0.01, and *** *p* < 0.001; otherwise, a non-significant difference is recorded.

To assess the effects of both single and continuous 7-day metformin injections on the established reserpine-induced thermal hypersensitivity in the male and female mice, we measured their responses using a hot plate test. The hot plate test evaluates an animal’s response to a painful thermal stimulus, allowing researchers to quantify their sensitivity to heat pain [35]. In the male mice, reserpine injections significantly reduced their latency to respond to heat stimulus compared to the vehicle control group in both the tested days (*p* = 0.0113 and *p* = 0.0131, respectively). Although a single dose of metformin did not produce an acute positive effect on their thermal hypersensitivity, metformin treatment for seven consecutive days significantly increased their latency to respond to heat stimulus compared to the saline-treated mice (*p* = 0.0486; Figure 3A,B).

Similar results were observed in the female mice as subcutaneous injections of reserpine significantly reduced their latency to respond to hot stimulus on days 4 and 10 compared to the vehicle control group (*p* = 0.0096 and *p* = 0.0158, respectively). Although a single injection of metformin did not affect the thermal hypersensitivity, continuous metformin treatment for seven consecutive days significantly increased their latency to respond to hot stimulus compared to saline-treated mice (*p* = 0.0134; Figure 3C,D).

In contrast to the effects of metformin, a single pregabalin injection significantly increased their latency to respond to hot stimuli compared to the saline group in both the male (*p* = 0.0075) and female mice (*p* = 0.0020).

### 3.2. Metformin Treatment Reversed Motor Defects in Male Mice Only 

While FM primarily manifests symptoms related to pain, some patients may also experience motor defects or impairments. One of the prevalent associated symptoms of FM is fatigue, which affects about 76% of FM patients [45]. Through motor tests, we aimed to assess fatigue in the mice. TDM and velocity tests evaluated spontaneous motor behavior, which can indirectly reflect fatigue-like symptoms. The rotarod test, on the other hand, measures forced motor activity, providing a more targeted assessment of fatigue. 

**Figure 4:**
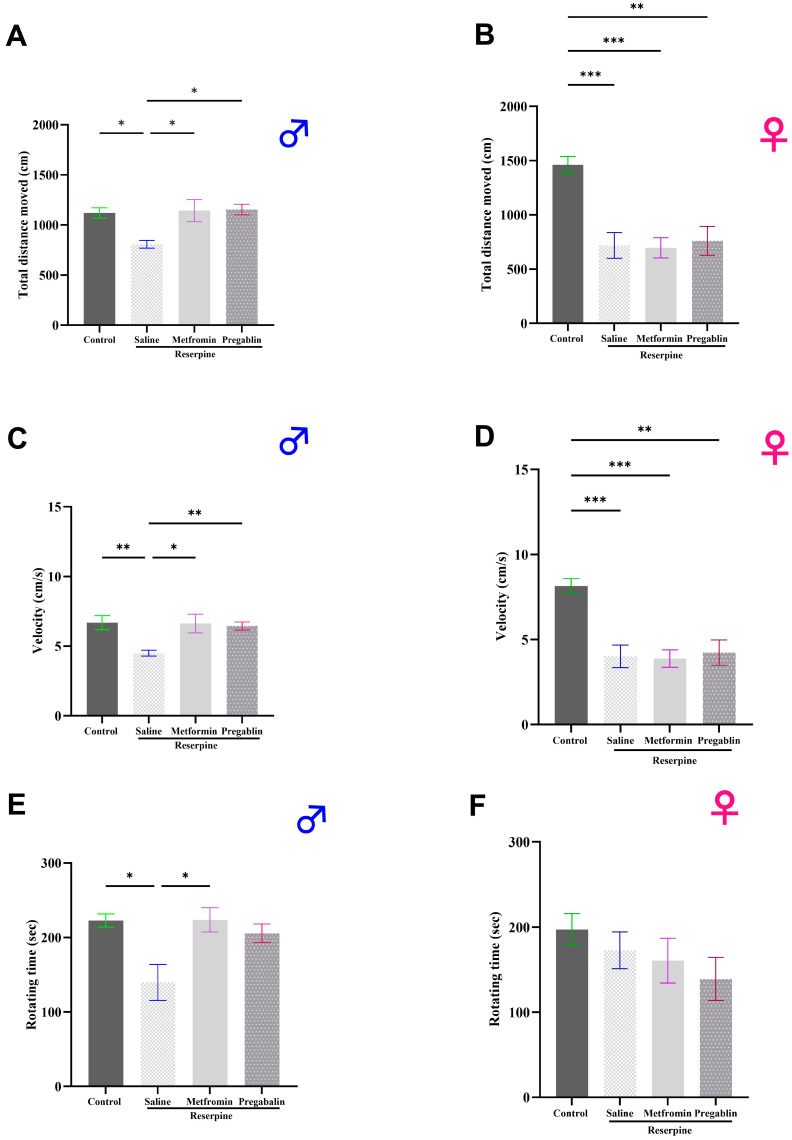
Effect of different interventions on motor performances in male and female mice. (**A**,**B**) Total distance moved (TDM) and (**C**,**D**) velocity of mice in open field test, performed on day 9 of study. Reserpine administration reduced TDM and velocity in both male and female mice. Metformin administration reversed effects of reserpine on TDM and velocity in male mice only. (**E**,**F**) Rotating time in rotarod test, conducted on day 7 of study. Reserpine administration reduced rotating time only in male mice, and metformin administration reversed that effect. Each bar represents mean, and vertical lines indicate standard error mean (SEM) for 6–12 mice/group. Asterisks above lines indicate a significant difference between groups where * *p* < 0.05, ** *p* < 0.01, and *** *p* < 0.001; otherwise, non-significant difference is recorded.

Therefore, the effects of reserpine and metformin on motor performance were evaluated through various behavioral tests in the male and female mice. We used the OFT to assess the TDM and velocity of the mice, therefore evaluating their spontaneous motor activity. Reserpine administration significantly reduced the TDM in both the male and female mice compared to the controls (*p* = 0.0308 and *p* = 0.0007, respectively). Metformin treatment reversed the reserpine-induced reduction in their TDM, but this effect was significant only in the male mice (*p* = 0.0149; Figure 4A,B). 

Reserpine administration also led to a significant decrease in the velocity in both the male and female mice during the OFT (*p* = 0.0092 and *p* = 0.0008, respectively). Metformin treatment successfully reversed the reduction in velocity caused by reserpine in male mice (*p* = 0.0285; Figure 4C), whereas no significant effect was observed in the female mice (Figure 4D).

In addition, the forced motor behavior was assessed in the mice using a rotarod test. Reserpine administration significantly reduced the rotating time only in the male mice compared to the vehicle control group (*p* = 0.0287), indicating fatigue and impaired motor coordination. Metformin treatment effectively reversed this reduction, restoring their normal motor performance (*p* = 0.0141; Figure 4E). However, the female mice showed no differences across all the groups in the rotarod test (Figure 4F).

### 3.3. Metformin Demonstrated Antidepressant-Like Effects in Male and Female Mice

**Figure 5:**
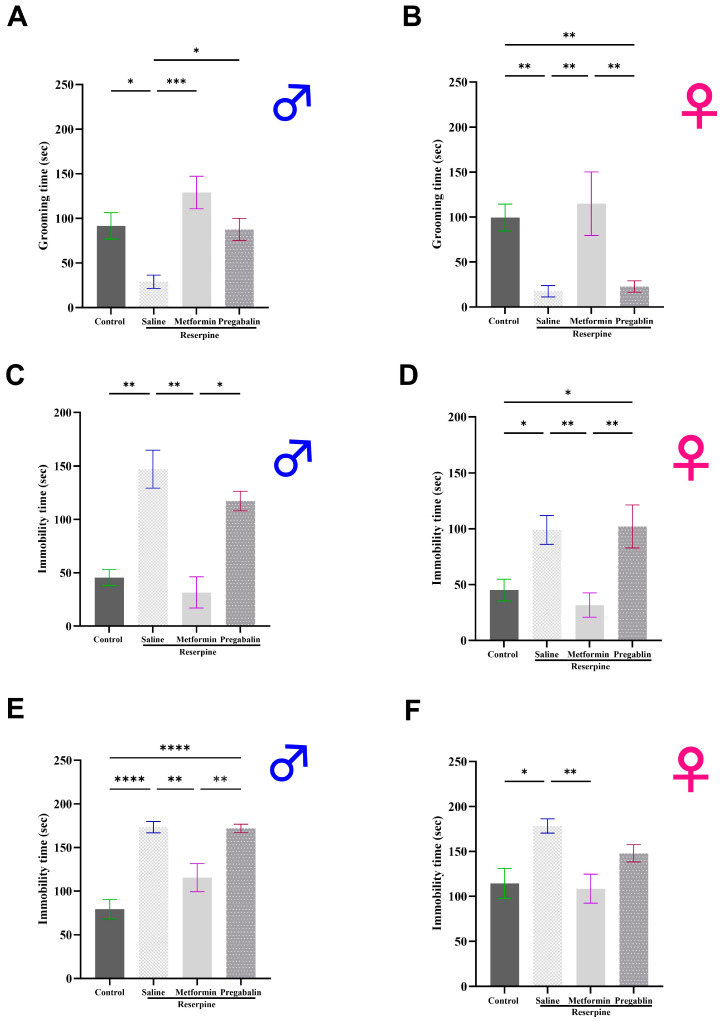
Effect of different interventions on depressive-like behavior in male and female mice. (**A**,**B**) Grooming time in splash test, conducted on day 7 of study. Reserpine injections significantly decreased grooming time in both male and female mice. Metformin treatment reversed decrease in both sexes. (**C**,**D**) Immobility time in forced swimming test, performed on day 8 of study. Reserpine injections significantly increased immobility time in both male and female mice. Metformin reversed increase in both sexes. (**E**,**F**) Immobility time in tail suspension test, performed on day 9 of study. Reserpine injections significantly increased immobility time in both male and female mice. Metformin treatment successfully reversed increase in both sexes. Notably, pregabalin administration had no significant effect in any tests except for splash test, where it showed effect only in male mice. Each bar represents mean, and vertical lines indicate standard error mean (SEM) for 8–12 mice/group. Asterisks above lines indicate significant difference between groups where * *p* < 0.05, ** *p* < 0.01, *** *p* < 0.001, and **** *p* < 0.0001; otherwise, non-significant difference is recorded.

Next, to explore the potential antidepressant-like effects of metformin, we assessed the depressive-like behaviors in the male and female mice using the ST, FST, and TST. Both the male and female mice displayed pronounced depression-like behavior after three days of reserpine injections, as shown in Figure 5. Metformin treatments significantly increased the grooming time in the ST compared to the saline group in both the male (*p* = 0.0002; Figure 5A) and female mice (*p* = 0.0036; Figure 5B). Moreover, metformin administration significantly reduced the total immobility time in the FST compared to the saline group in both the male (*p* = 0.0014; Figure 5C) and female mice (*p* = 0.0047; Figure 5D). Similarly, metformin administration significantly reduced the total immobility time in the TST compared to the saline group in both the male (*p* = 0.0017; Figure 5E) and female mice (*p* = 0.0059; Figure 5F). Interestingly, metformin’s effects surpassed those of pregabalin, as pregabalin treatment did not improve the depression-like behavior in the male and female mice across all the tests, except for its impacts on the grooming time during the ST in the male mice.

### 3.4. Metformin Modulated Neurotransmitter Levels in the Brain and Spinal Cord of Male Mice, but Not Females 

**Figure 6:**
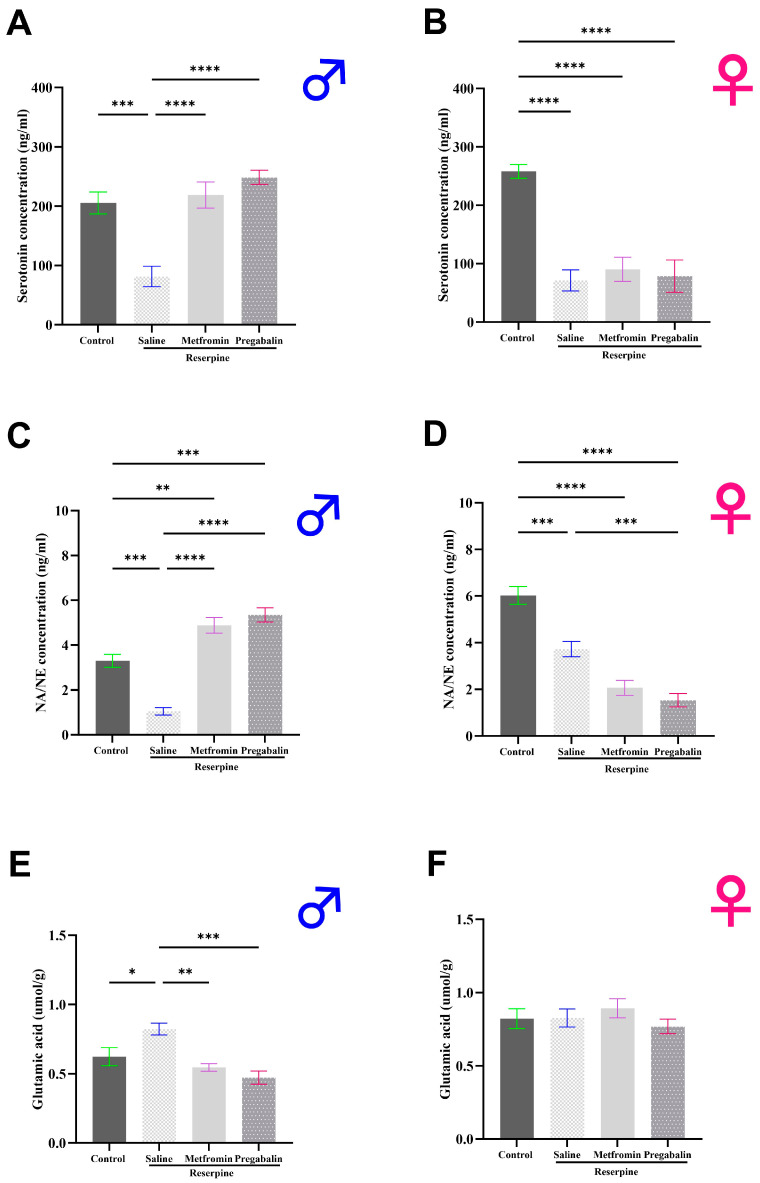
Effect of different interventions on neurotransmitter levels in total brains of male and female mice. (**A**,**B**) Serotonin levels: reserpine injections significantly decreased serotonin levels in both male and female mice. Metformin treatment reversed decrease in male mice only. (**C**,**D**) Norepinephrine levels: reserpine injections significantly decreased norepinephrine levels in both male and female mice. Metformin reversed decrease in male mice only. (**E**,**F**) Glutamate levels: reserpine injections significantly increased glutamate levels in brains of male mice, and metformin treatment reversed increase. In female mice, no significant differences were observed between any groups. Each bar represents mean, and vertical lines indicate standard error mean (SEM) for 6–8 mice/group. Asterisks above lines indicate significant difference between groups where * *p* < 0.05, ** *p* < 0.01, *** *p* < 0.001, and **** *p* < 0.0001; otherwise, a non-significant difference is recorded.

Following behavioral tests, we dissected the animals and collected their brains and spinal cords to measure their neurotransmitter levels (day 11). In the male mice, the serotonin and norepinephrine levels significantly decreased while the glutamate levels significantly increased following reserpine administration compared to the vehicle control group (*p* = 0.0003, *p* = 0.0002, and *p* = 0.0488, respectively). Metformin treatment restored the levels of serotonin and norepinephrine toward the control, therefore revealing significant differences compared to the RES + saline group (*p* < 0.0001; Figure 6A,C). In addition, metformin significantly reduced the glutamate levels compared to the RES + saline group (*p* = 0.0059; Figure 6E). 

The results in the female mice were markedly different. Reserpine administration significantly decreased the serotonin and norepinephrine levels in the female mice compared to the vehicle control group (*p* < 0.0001, and *p* = 0.0002, respectively). However, the metformin and pregabalin treatments failed to improve the serotonin and norepinephrine levels in the brains of the female mice (Figure 6B,D). No significant differences were observed in the glutamate levels among the female mice groups (Figure 6F).

**Figure 7:**
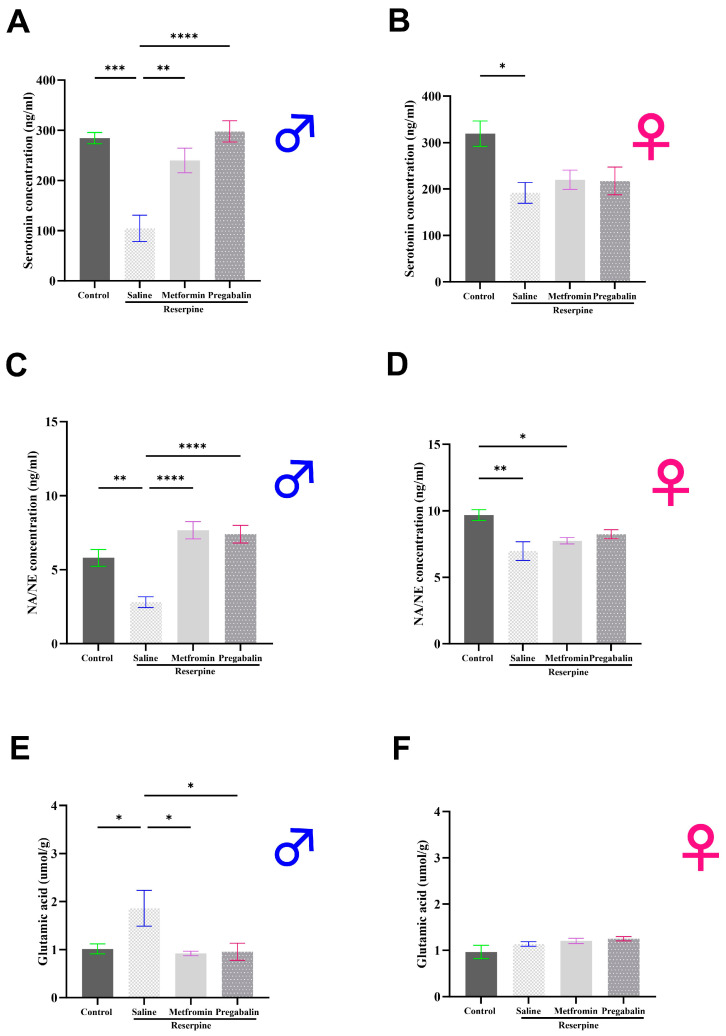
Effect of different interventions on neurotransmitter levels in spinal cord of male and female mice. (**A**,**B**) Serotonin levels: reserpine injections significantly decreased serotonin levels in both male and female mice. Metformin treatment reversed decrease in male mice only. (**C**,**D**) Norepinephrine levels: reserpine injections significantly decreased norepinephrine levels in both male and female mice. Metformin treatment reversed decrease in male mice only. (**E**,**F**) Glutamate levels: reserpine injections significantly increased glutamate levels in spinal cords of male mice, and metformin treatment reversed increase. No differences were observed between any groups in female mice. Each bar represents mean, and vertical lines indicate standard error mean (SEM) for 5–6 mice/group. Asterisks above lines indicate a significant difference between groups where * *p* < 0.05, ** *p* < 0.01, *** *p* < 0.001, and **** *p* < 0.0001; otherwise, a non-significant difference is recorded.

The results observed in the spinal cord closely mirrored those found in the brain. In the male mice, the serotonin and norepinephrine levels significantly decreased in the RES + saline group compared to the vehicle control group, while the glutamate levels significantly increased (*p* = 0.0003, *p* = 0.0046, and *p* = 0.0393, respectively). Metformin treatment significantly alleviated the reductions in the serotonin and norepinephrine levels and reduced the elevated glutamate levels compared to the RES + saline group (*p* = 0.0029, *p* < 0.0001, and *p* = 0.0267, respectively; Figure 7A,C,E).

Consistent with the brain findings, the spinal cord results in the female mice were markedly different from those in the male mice. Both the serotonin and norepinephrine levels were reduced significantly in the reserpine-injected mice compared to the vehicle control group (*p* = 0.0160 and *p* = 0.0023, respectively). Metformin administration had no effect on the neurotransmitter levels in the spinal cord of the female mice (Figure 7B,D,F).

### 3.5. Metformin Modulated IL-1β Levels in the Brain and Spinal Cord of Both Male and Female Mice

**Figure 8:**
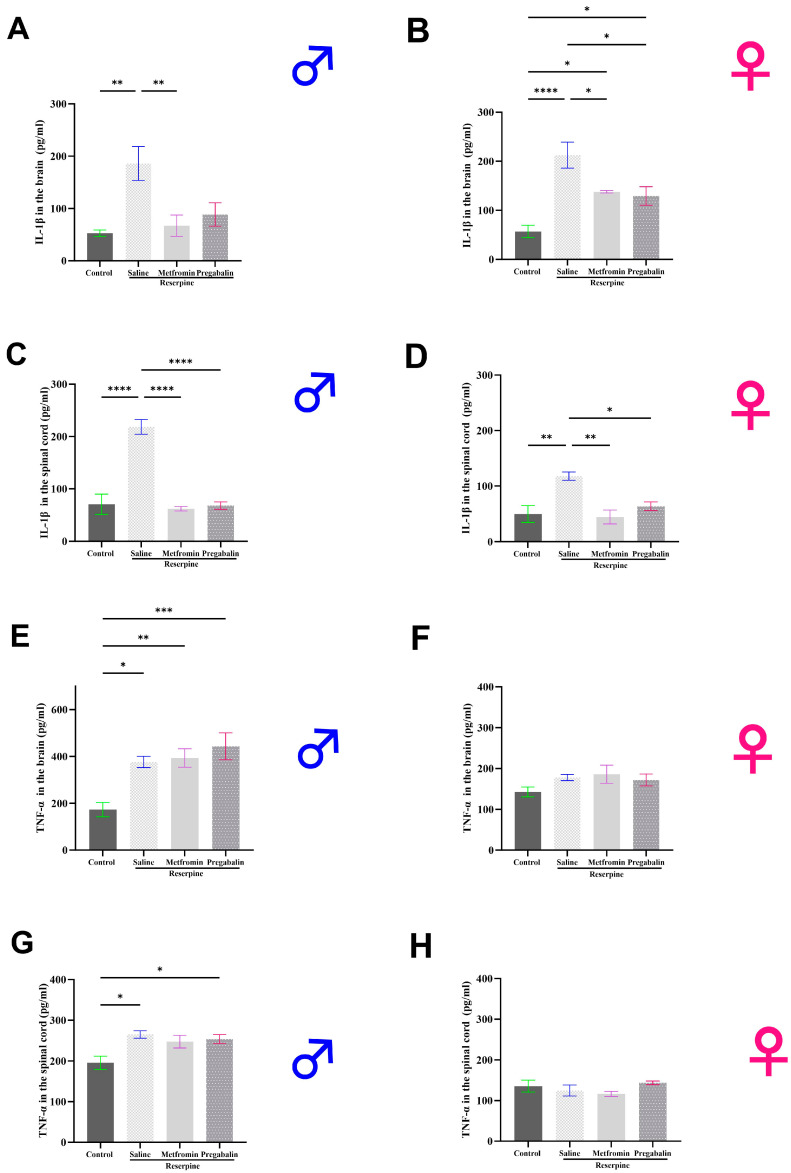
Effect of different interventions on proinflammatory cytokine levels in the total brain and spinal cord of male and female mice. (**A**,**B**) IL-1β levels in the brain: reserpine injections significantly increased IL-1β levels in both the male and female mice. Metformin treatment reversed this decrease in both the male and female mice. (**C**,**D**) IL-1β levels in the spinal cord: reserpine injections significantly increased the IL-1β levels in both the male and female mice. Metformin treatment reversed this decrease in both the male and female mice. (**E**,**F**) TNF-α levels in the brain: reserpine injections significantly increased the TNF-α levels in the male mice only. Metformin treatment reversed this increase in the male mice. (**G**,**H**) TNF-α levels in the spinal cord: reserpine injections significantly increased the TNF-α levels in the male mice only. However, metformin did not reverse this increase. Each bar represents the mean, and the vertical lines indicate the standard error mean (SEM) for the 4–6 mice/group. Asterisks above the lines indicate a significant difference between the groups where * *p* < 0.05, ** *p* < 0.01, *** *p* < 0.001, and **** *p* < 0.0001; otherwise, a non-significant difference is recorded.

In addition to measuring neurotransmitter levels, the levels of the proinflammatory cytokines in the male and female mice were also measured. It was observed that the IL-1β levels were significantly elevated in the brain and spinal cord of both the male and female RES + saline groups compared to the vehicle control group. Metformin treatment significantly decreased the IL-1β levels in the brain of both the male and female mice compared to the RES + saline group (*p* = 0.0063 and *p* = 0.0259, respectively; Figure 8A,B). Similarly, metformin treatment significantly decreased the IL-1β levels in the spinal cord of both the male and female mice compared to the RES + saline group (*p* < 0.0001 and *p* = 0.0033, respectively) (Figure 8C,D). The TNF-α levels also increased significantly in the brain (Figure 8E) and spinal cord (Figure 8G) of the male RES + saline groups compared to the vehicle control group (*p* = 0.0146 and *p* = 0.0101, respectively). Although, metformin treatment did not affect the TNF-α levels in the male mice in either the brain or spinal cord. In the female mice, no difference was detected between any of the groups in the TNF-α levels in either region (Figure 8F,H).

### 3.6. Metformin Alleviated Reserpine Induced-Histopathological Changes in the Hippocampus, Thalamus, and Spinal Cord 

**Figure 9:**
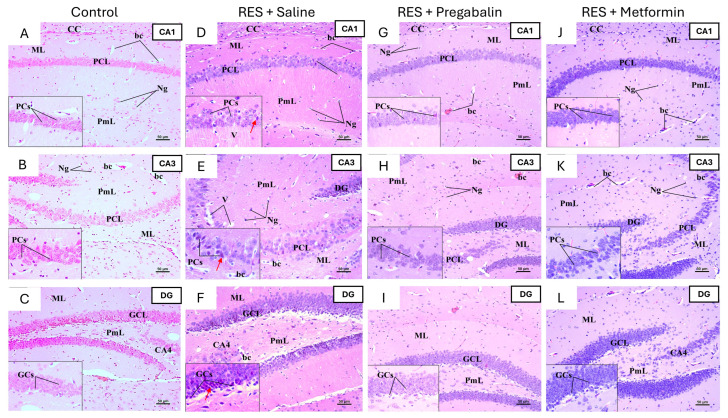
Representative photomicrographs of H&E-stained hippocampus sections in male mice following metformin treatment. (**A**,**B**) Control group: CA1 and CA3 regions of control group displayed all layers—outer molecular layer (ML), middle pyramidal cell layer (PCL), and inner polymorphic layer (PmL)—with normal morphology. PCL comprised well-organized pyramidal cells (PCs) containing large vesicular nuclei and pale basophilic cytoplasm. (**C**) Dentate gyrus (DG) was formed by upper and lower limbs, each consisting of three layers: ML, granular cell layer (GCL), and PmL. Inset of upper limb showed that GCL was composed of densely packed, rounded granule cells (GCs). (**D**,**E**) RES + saline group: CA1 and CA3 regions exhibited disorganized PCL compared to control group. Most PCs appeared shrunken, with dark-stained cytoplasm, ill-defined nuclei, and pericellular halos (red arrow). Both ML and PmL contained an increased number of neuroglial cells (Ng), variable-sized vacuoles (V), and dilated blood capillaries (bc). (**F**) DG exhibited several degenerated and shrunken GCs (red arrow). (**G**,**H**) RES + pregabalin group: CA1 and CA3 regions showed improvement, appearing more similar to control group. Many PCs displayed a normal appearance with vesicular nuclei, although some PCs appeared condensed with dark basophilic cytoplasm. Both ML and PmL contained more Ng cells and dilated bc. (**I**) Architectural improvements were observed in GCL, although some granular cells still appeared shrunken with condensed nuclei. (**J**,**K**) RES + metformin group: CA1 and CA3 regions demonstrated improved appearance, closely resembling control group. However, some PCs with dark basophilic cytoplasm and unclear nuclei were still present. (**L**) DG structure showed significant improvement, appearing nearly identical to control group. Inset of GCL contained densely packed, rounded-to-oval GCs without signs of degeneration. CC, Corpus Callosum. Scale bar corresponds to 50 µm (H&E × 200, Inset × 400).

**Figure 10:**
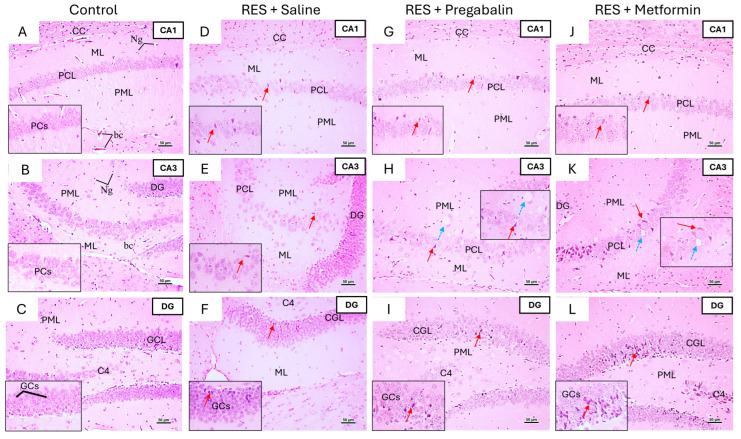
Representative photomicrographs of H&E-stained hippocampus sections in female mice following metformin treatment. (**A**,**B**) Control group: CA1 and CA3 regions displayed normal pyramidal cells (PCs) with large, pale vesicular nuclei. (**C**) Dentate gyrus (DG) of control group exhibited normal granular cells (GCs) with vesicular nuclei in granular cell layer (GCL). (**D**,**E**) RES + saline group: CA1 and CA3 regions in group showed several degenerated, dark, shrunken PCs with dark, ill-defined shaped nuclei (red arrow). (**F**) DG exhibited several degenerated and shrunken GCs (red arrow). (**G**–**I**) RES + pregabalin group: pregabalin treatment did not alleviate toxic effects of reserpine, as several degenerative changes were still evident following treatment. (**J**–**L**) RES + metformin group: metformin treatment also failed to mitigate toxic effects of reserpine across all areas, as degenerative changes persisted and tissue vesiculation was observed in polymorphic layer (PML) (light blue arrow). bc, Blood Capillary; CC, Corpus Callosum; ML, molecular layer; PCL, pyramidal cell layer. Scale bar corresponds to 50 µm (H&E × 200, Inset × 400).

To further assess the impact of metformin on the regions involved in pain perception, histological examinations of the hippocampus, thalamus, and spinal cord were conducted using H&E staining. The hippocampus is typically structured with the Cornu Ammonis (composed of CA1, CA2, CA3, and CA4) and the dentate gyrus. In the control group of both the male and female mice, the CA1, CA3, and dentate gyrus regions displayed normal morphology (Figure 9 and Figure 10). In the RES + saline group of the male mice, the hippocampus exhibited alterations across the CA1, CA3, and dentate gyrus regions, including degenerative changes in the pyramidal and granular cell layers. Pregabalin treatment ameliorated these histopathological alterations, with most pyramidal cells preserving their morphology and displaying vesicular nuclei. Additionally, the granular cell layer demonstrated improvements. Interestingly, the metformin treatment resulted in significant histopathological improvements compared to the RES + saline group, with the CA1, CA3, and dentate gyrus regions appearing comparable to the control group (Figure 9).

Similar degenerative changes were noticed in the hippocampus of female mice following reserpine administration, including pyramidal cell degeneration, shrunken neurons with dark basophilic cytoplasm, and poorly defined nuclei in the pyramidal cell layers of CA1 and CA3. The degeneration of granular cells was also evident in the granular cell layer of the dentate gyrus. However, metformin and pregabalin treatments did not demonstrate significant improvements compared to the RES + saline group across all the regions (Figure 10).

**Figure 11:**
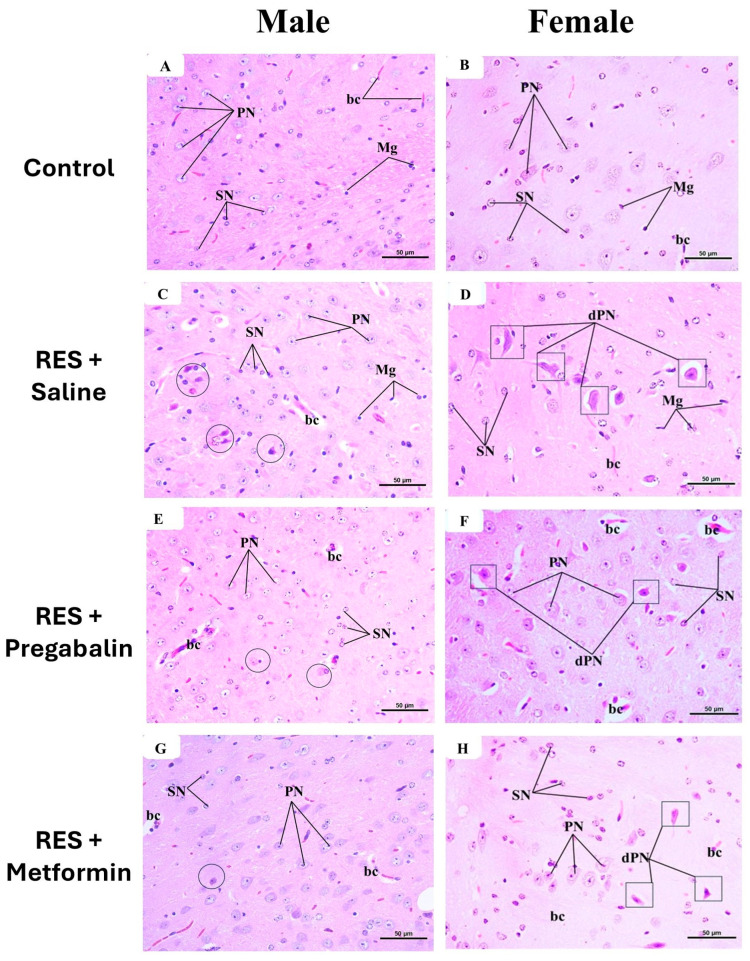
Representative photomicrographs of H&E-stained thalamus sections in male and female mice following metformin treatment. (**A**,**B**) Control group exhibited normal thalamic structure, featuring large principal neurons (PNs) and small neurons (SN). Numerous microglial cells (Mg) and tiny capillaries (bc) were also observed. (**C**,**D**) Reserpine (RES) + saline group displayed numerous degenerated principal neurons (dPNs), marked by black circles in male group and black squares in female group. (**E**,**F**) RES + pregabalin group also exhibited structure similar to control group but with some degenerated PNs, marked by black circles in male group and black squares in female group. (**G**,**H**) RES + metformin group showed a structure that closely resembled control group, though fewer degenerated PNs were still observed, marked by black circles in the male group and black squares in the female group. The scale bar corresponds to 50 µm (H&E × 400).

Figure 11A,B presents the histological structure of the thalamus in the control group for both the male and female mice, which appears normal. The neuropil exhibited a multitude of healthy neurons of various types within a pinkish background. The predominant cell type observed was the principal cell, characterized by large, pale, spherical nuclei. Additionally, small cells with dark nuclei were also observed. Numerous microglial cells with small, dark nuclei and surrounding halos, along with abundant nerve fibers and tiny capillaries, were present within the neuropil. In the RES + saline-treated group, as depicted in Figure 11C,D, many principal cells and some small cells appeared degenerated and condensed compared to the control group. Figure 11E,F illustrates the thalamus from the pregabalin-treated group, showing a structure that closely resembled that of the control group, with fewer degenerated principal cells and some dilated capillaries. Metformin administration decreased the appearance of the degenerated principal cells compared to the reserpine-treated group in the male mice (Figure 11G), while the neuroprotective effects of metformin were lesser in the female mice than those seen in male mice (Figure 11H). 

**Figure 12:**
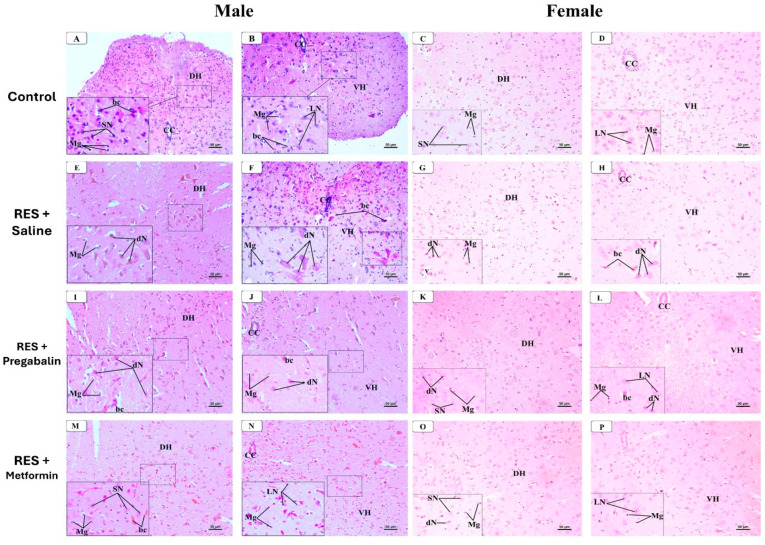
Representative photomicrographs of H&E-stained spinal cord sections in male and female mice following metformin treatment. (**A**–**D**) Control group exhibited dorsal horn of gray matter (DH) containing small multipolar neurons (SN) and ventral horn (VH) containing large multipolar neurons (LN). Microglial cells (Mg) and tiny capillaries were observed dispersed in both horns. (**E**–**H**) RES + saline group showed numerous degenerated neurons (dNs) in both dorsal and ventral horns, characterized by dark eosinophilic cytoplasm and condensed nuclei. Various-sized vacuoles and dilated capillaries were observed in neuropil. (**I**–**L**) RES + pregabalin group displayed fewer dNs in both dorsal and ventral horns. However, some vacuoles and dilated capillaries remained in neuropil. (**M**–**P**) RES + metformin-treated group showed nearly normal neurons in both dorsal and ventral horns, except for some congested capillaries. CC, central canal. Scale bar corresponds to 50 µm (H&E × 200, Inset × 400).

In the spinal cord (Figure 12), the examination of the H&E-stained sections at the mid-thoracic region from the control group of both the male and female mice revealed a normal histological structure. The dorsal and ventral horns of the gray matter contained various types of neurons and microglial cells within an eosinophilic neuropil matrix, along with tiny blood capillaries. The dorsal horn neurons were predominantly small multipolar neurons with small nuclei and dark cytoplasm, while the ventral horn neurons were mainly large multipolar neurons with large vesicular nuclei, basophilic cytoplasm, and prominent nucleoli (Figure 12A–D). In the RES + saline-treated male and female groups, the dorsal horn exhibited several degenerated small neurons with dark eosinophilic cytoplasm and condensed nuclei. Many large neurons in the ventral horn also appeared degenerated, characterized by dark, acidophilic cytoplasm and condensed nuclei. Both the horns exhibited various-sized vacuoles and dilated capillaries (Figure 12E–H). Pregabalin administration resulted in fewer degenerated neurons in both the dorsal and ventral horns compared to the saline-treated group in both the male and female mice. However, some vacuoles and dilated capillaries were still observed in the neuropil (Figure 12I–L). Furthermore, metformin administration to the male and female mice resulted in nearly normal neurons in both the dorsal and ventral horns, with only the irregular appearance of some congested capillaries (Figure 12M–P).

## 4. Discussion

The current pharmacotherapy for FM has limited efficacy and varies among individuals, with responses to medications often being subjective [46]. Additionally, the chronic nature of FM pain necessitates continuous long-term treatment, sometimes extending up to 176–205 days, while pregabalin, a first-line approved drug for FM, has a potential for abuse [47]. These factors underscore the critical need for research aimed at identifying novel therapeutic strategies for FM. In this study, the effects of metformin administration on reserpine-induced FM in male and female mice were assessed. Reserpine, known for its ability to deplete monoamine neurotransmitters, led to a significant decrease in the serotonin and norepinephrine levels in both the brain and spinal cord [26]. Furthermore, reserpine administration increased the glutamate levels in these regions, indicating potential excitotoxicity and neuronal damage [27]. These effects established an FM-like model in the rodents, as observed in the present study [48]. 

While the pain-relieving effects of metformin have been shown in other pain models, this study is, to the best available knowledge, the first to demonstrate its potential benefits in a model of FM in mice. The key finding is that metformin demonstrates a promising effect on FM-like pain after seven days of continuous administration in both male and female mice, as evidenced by its ability to increase the paw withdrawal threshold in a vFt test and increase the latency to respond to hot stimulus in a hot plate test. It has been proven that metformin interacts with crucial molecules that significantly contribute to nociceptive processing. Previous studies have described metformin’s ability to ease pain in animal models through its ability to activate AMP-activated protein kinase (AMPK), which plays a fundamental role in controlling neural excitability [17,49]. Metformin has been found to activate the opioidergic pathways in models of nociceptive and nerve pain [50]. Metformin also prevents incision-evoked mechanical hypersensitivity and hyperalgesia [51]. It also reverses and prevents the complete occurrence of neuropathic pain, and this has a negative correlation with microglial activity in the spared nerve injury model [19].

However, a single metformin injection did not enhance reserpine-induced mechanical or thermal hyperalgesia, either in the male or female mice. According to the existing literature, the effective dose and duration of metformin in modulating the key molecules involved in nociceptive processing remain subject to debate. For instance, the oral delivery of metformin at doses of 180 mg/kg and 250 mg/kg has been shown to reduce nociceptive responses within half an hour of administration [52]. Other studies support the findings of this study, indicating that a single dose of metformin fails to produce acute analgesia, and the antinociceptive effects of metformin take several days to express [53,54]. 

The behavioral assessments of the motor activity in male mice indicated that reserpine-induced motor impairment notably improved after metformin administration. Metformin’s neuroprotective effects and influence on neurotransmitter levels, as we will discuss later, may contribute to an improvement in motor activity, enhancing synaptic transmission and neural plasticity. In parallel to this study’s findings, George Jitica and his colleagues demonstrated metformin’s ability to improve motor coordination in the rotarod test following impairment induced by haloperidol, corroborating our findings [55]. Moreover, metformin increases the expression of brain-derived neurotrophic factor (BDNF), a protein that aids in the development, maintenance, and survival of neurons [56]. BDNF is also involved in neuroplasticity, which is vital for learning and memory. Improved insulin sensitivity, another benefit of metformin, has been correlated with better mental health outcomes. Insulin resistance has been linked to the etiology of depression, and metformin’s potential to improve insulin sensitivity might contribute to its antidepressant effects [57]. In addition, the anti-inflammatory properties of metformin may help to improve mood and cognitive function [58]. The findings of this study align with these results, showing a significant reduction in the depressive-related behavior in the mice following metformin administration. Additionally, pregabalin was used as a positive control drug, given its FDA approval for treating FM in this study. While clinical trials have shown pregabalin’s effectiveness in alleviating FM-related pain, it does not appear to exhibit antidepressant effects in individuals with FM [59]. In our study, pregabalin administration did not improve the depressive-like behavior in the mice during the TST and FST. These results highlight metformin’s potential superiority over pregabalin for treating FM, as depression is a strongly associated symptom with chronic pain in FM [60].

Serotonin, norepinephrine, and glutamate neurotransmitters are critical for pain modulation, mood regulation, and sleep, and their dysregulation likely contributes to heightened pain perception and other symptoms experienced by individuals with FM [61,62]. Serotonin plays a key role in modulating pain perception and mood; its dysregulation may contribute to the sensory hypersensitivity and depressive symptoms commonly observed in FM patients [63,64]. Norepinephrine is crucial for descending pain modulation pathways, and its alterations can exacerbate pain severity and fatigue in FM [65]. Glutamate, the primary excitatory neurotransmitter in the central nervous system, is associated with amplifying pain signals and promoting neuroinflammation, potentially contributing to the chronic pain and hypersensitivity characteristic of FM [66]. One of the critical findings in our experiments is that metformin administration restored the serotonin and norepinephrine levels to near-normal values in the brain and spinal cord of male mice. Moreover, metformin reduced the glutamate levels, suggesting its role in mitigating excitotoxic processes. Consistent with our findings, metformin supplementation significantly ameliorated the serotonin and norepinephrine levels in the brains of rodents [22,23]. Furthermore, metformin administration reduced the brain glutamate to normal levels in a diabetic epileptic model [24]. While preclinical studies have highlighted metformin’s ability to modify neurotransmitter levels, most research has focused on its effects on depression, particularly as a comorbid symptom of diabetes. This makes our study a pioneer in uncovering metformin’s possible impact on neurotransmitter alterations in FM. 

Besides neurotransmitter imbalance, nociceptive activation in FM has been linked to increasing levels of proinflammatory cytokines, including IL-1β and TNF-α [67]. Given metformin’s known anti-inflammatory properties, its potential for modulating IL-1β and TNF-α levels was assessed in this study, which could be another promising mechanism for metformin to alleviate FM-related nociceptive activation [68]. In this study, metformin significantly reduced the IL-1β levels in both the brain and spinal cord to near-normal levels in both male and female mice. AMPK activation can enhance cellular metabolism and reduce oxidative stress. It also inhibits the NLRP3 inflammasome and the nuclear factor-kappa B (NF-κB) signaling pathway, thereby reducing IL-1β production [69,70]. However, it is noteworthy that metformin did not affect the TNF-α levels in the brain or the spinal cord in either the male or female mice. The lack of effect on TNF-α suggests that other regulatory mechanisms may control the production of this cytokine or that strong doses or prolonged treatment duration may be required to observe changes in TNF-α levels.

This study also explored the prophylactic effects of metformin on the histopathological changes induced by a reserpine model of FM. To achieve this, the hippocampus, thalamus, and spinal cord of the male and female mice were examined using H&E staining. Changes in hippocampal structure and function are often observed in chronic pain conditions, underscoring their importance in understanding and treating pain [71]. This study revealed significant degenerative changes in multiple hippocampal layers following reserpine administration. These histological alterations align with previous findings from an FM rat model induced by acidic saline [72]. Clinical studies on FM patients reveal hippocampal changes, including metabolic dysfunction and volume reduction, which may explain sleep disturbances and cognitive dysfunction [73,74]. In this study, metformin administration effectively reversed the histopathological changes in the hippocampus associated with reserpine administration in male mice. Consistent with present study’s findings, metformin has been shown to correct the morphological state of neurons under specific pathological conditions. It enhances neuroplasticity, increases neuronal cell numbers, improves neuron survival, and reduces neuroinflammation in the CA1 and DG of rodents, thereby improving cognitive function and overall hippocampal health [75,76,77].

Histological changes in the thalamus resulting from reserpine administration are infrequently documented in the literature. In this study, reserpine administration led to significant changes in the thalamus of both the male and female mice, characterized by neuronal degeneration, vacuolation, and dilated capillaries. These histopathological alterations reflect the severe impact of reserpine on neural tissue integrity, contributing to the observed symptoms of FM in this experimental model, as documented in previous studies [78]. Interestingly, the findings of this study highlight metformin’s potential to prevent the structural damage induced by reserpine in the thalamus, which was more obvious in male mice.

Moreover, the spinal cord is pivotal in the modulation and transmission of pain signals, acting as a primary relay center that processes nociceptive information from peripheral nerves and transmits it to the brain [79]. This study observed neuronal degeneration in the dorsal and ventral horns of the spinal cord, along with the presence of vacuoles of various sizes and dilated capillaries in the neuropil following reserpine administration. These alterations may be linked to glutamatergic excitotoxicity following reserpine exposure [80]. Remarkably, metformin administration preserved the integrity of the spinal cord, highlighting its protective effects at this level. A previous study noted that the FM induction model using reserpine resulted in morphological changes in the motor neurons within the ventral horn of the spinal cord, whereas such changes were not observed in the dorsal horn [78]. Additionally, other studies suggest that reserpine can influence spinal cord function and alter neurotransmitter levels which may indirectly affect its histological features [81]. 

A crucial observation in this study is that metformin has intriguing sex-dependent effects on the neurotransmitter levels in both the brain and the spinal cord. In contrast to its effect on the male mice, metformin injections were unable to equalize the serotonin and norepinephrine levels in the female mice. Metformin is a water-soluble drug that crosses cell membranes using active transporters such as an organic cation transporter (OCT-2) [82]. It has been found that testosterone can influence the expression and activity of the OCT-2 receptor [83]. Higher androgen levels in males can lead to increased OCT-2 expression [84]. Therefore, the differences in OCT-2 expression between males and females may impact the pharmacokinetics of drugs that rely on this transporter, leading to differences in drug efficacy between the sexes, as found in this study. Sex-specific differences in the effect of metformin have been described by Kufreobong and colleagues. In their study, metformin was able to prevent neuropathic pain in the male mice but not in the female mice [19]. In another study, metformin administration led to distinct changes in the signaling pathways related to AMPK and autophagy, which are critical for cellular energy balance and inflammatory responses. These changes differed significantly between the male and female subjects, contributing to differences in the therapeutic outcomes [85]. Moreover, accumulated evidence indicates significant sex differences in the expression and activity of blood–brain barrier (BBB) transporters, with gonadal hormones playing a further modulatory role [86]. Therefore, the ability of metformin to cross the BBB may differ between males and females. These differences may significantly influence the drug’s effects on central nervous system processes, including the regulation of neurotransmitters. Hence, the observed inability of metformin to equalize serotonin and norepinephrine levels in female mice, despite its effects in male mice, may be partly due to these differences in BBB permeability and transporter expression. It is also important to mention that the symptoms of FM are influenced by the menstrual cycle and significant hormonal changes, such as those experienced during pregnancy and menopause [87]. These hormonal fluctuations can exacerbate symptoms, highlighting the importance of considering hormonal factors when studying FM, especially in female populations. This aspect may also provide insight into the limited efficacy of metformin in inducing full benefits in female mice, as hormonal variations could play a role in the response to treatment.

## 5. Strengths and Limitations

In regard to the strength of this study, this study offers valuable insights into the potential of metformin as a treatment for FM, with a detailed assessment of the neurotransmitter levels and histopathological changes in a well-established FM mouse model. The inclusion of both male and female mice allowed for an exploration of the sex-specific differences in the treatment effects.

Despite these strengths, this study has certain limitations. It relies on a single animal model and features a relatively short treatment duration, which may not fully capture the long-term therapeutic potential of metformin. Additionally, while a histopathological analysis was conducted, more detailed tissue examination, particularly at the cellular and molecular levels, is necessary to further elucidate metformin’s neuroprotective effects. Future studies should incorporate extended treatment periods, the use of multiple FM models, and a more thorough investigation of tissue integrity, including advanced histological techniques, to provide a deeper understanding of the drug’s effects on neural and musculoskeletal tissues. 

## 6. Conclusions

In this study, metformin effectively alleviated mechanical and thermal hyperalgesia, improved depressive-like behaviors, reduced IL-1β levels, and prevented structural damage in the spinal cord in both male and female mice. These findings suggest that metformin could be a possible supportive agent candidate for future clinical trials aimed at alleviating hyperalgesia and depressive symptoms in FM. Importantly, the sex-specific differences observed in the neurotransmitter regulation of metformin underscore the necessity of including sex-specific analyses in its mechanism of action in the conditions associated with neurotransmitter disturbances such as FM. However, while metformin appeared to modulate monoamine levels, it is important to consider that these changes may be region-specific within the brain, potentially leading to varied behavioral outcomes, meriting further research and clinical evaluation. 

## Data Availability

The data will be available upon request.
